# UBL3 Participates in the Early Stages of CD83‐Dependent CD4^+^ T Cell Selection

**DOI:** 10.1002/eji.70143

**Published:** 2026-02-04

**Authors:** Huw Morgan, Haiyin Liu, Jose A. Villadangos, Justine D. Mintern

**Affiliations:** ^1^ Department of Biochemistry and Pharmacology, Bio21 Molecular Science and Biotechnology Institute The University of Melbourne Parkville Victoria Australia; ^2^ Department of Microbiology and Immunology, Peter Doherty Institute for Infection and Immunity The University of Melbourne Parkville Victoria Australia

## Abstract

CD83 is critical for CD4^+^ T cell selection. It regulates MHC II ubiquitination and turnover at the surface of thymic epithelial cells (TECs). The role of UBL3, a recently identified adaptor molecule for MHC II ubiquitination, is unknown in thymic selection. Here we demonstrate that UBL3 regulates MHC II in TECs and participates in CD4^+^ T cell selection. Deleting UBL3 in CD83 loss‐of‐function mice (*Cd83^anu/anu^ Ubl3^−/−^*) increases MHC II on the surface of *Cd83^anu/anu^* TECs. This increase in surface MHC II correlates with increased positive selection of CD4^+^ T cells. Analysis of *Cd83^anu/anu^* and *Cd83^anu/anu^ Ubl3^−/−^* mice identifies the CD4^+^ CD8^low^ CD69^+^ stage of positive selection as the origin of the CD4^+^ T cell selection defect in *Cd83^anu/anu^* mice. This stage of CD4^+^ T cell positive selection is also impacted by UBL3. The positive selection defect in the absence of CD83 also manifests as alterations in CCR7^+^ CD4 single‐positive (SP) thymocytes. At the later stages of CD4^+^ T cell development, a role for UBL3 is no longer detected. In summary, through in‐depth phenotyping of thymocyte populations, a role for CD83 and UBL3 in regulating the early stages of CD4^+^ T cell positive selection has been identified.

## Graphical abstract

CD83 and UBL3 are regulators of MHCII surface expression; however, only CD83 has a known role in regulating CD4^+^ T cell selection. This paper describes a role for UBL3 in regulating MHC II expression on thymic epithelium in a CD83‐deficient context, leading to alterations in CD4^+^ T cell positive selection.

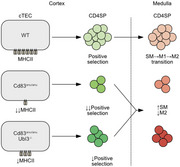

## 1 Introduction

During thymocyte development, thymic selection generates a T cell repertoire poised to eliminate foreign material with minimal reactivity to self. Positive selection occurs when CD4^+^CD8^+^ (double positive, DP) thymocytes are committed to the CD8 or CD4 T cell lineage based upon viable T cell receptor (TCR) engagement with MHC I or MHC II, respectively. Negative selection deletes potentially harmful auto‐reactive thymocytes that express TCRs with high affinity for self‐antigen. Positive and negative selection are typically stratified in the cortical and medullary regions of the thymus, where distinct populations of antigen‐presenting thymic epithelial cells (TECs) reside [1].

MHC II is critical for CD4^+^ T cell selection. An important determinant of MHC II expression is its turnover at the plasma membrane. A major mechanism by which this is regulated is ubiquitination, where a poly‐ubiquitin (Ub) chain is covalently attached to the cytosolic tail of MHC II [2, 3]. Ubiquitination promotes MHC II traffic away from the plasma membrane, where it is degraded in lysosomes [2]. MHC II ubiquitination in cortical TEC (cTEC) and medullary TEC (mTEC) is regulated by the E3 Ub ligase MARCH8 [4, 5]. CD83, a transmembrane protein, also participates in this response and acts as a brake to limit unwarranted MARCH8 activity. Loss of functional CD83 in mice results in significantly impaired CD4^+^ T cell selection that is likely due to the disproportionate MARCH8‐mediated MHC II ubiquitination, elevated MHC II turnover, and reduced MHC II at the surface of TECs. This is evidenced by significantly reduced numbers of splenic CD4^+^ T cells in the absence of functional CD83, a phenotype that can be restored upon deletion of MARCH8 [4, 5].

UBL3 is a newly identified adaptor molecule critical for MHC II ubiquitination and turnover in haemopoietic cells [6]. UBL3 participates in ubiquitination by MARCH1, a close relative of MARCH8. It is currently unknown whether UBL3 also regulates ubiquitination by MARCH8 and thereby participates in thymic MHC II turnover and CD4^+^ T cell selection. This is expected to be the case due to the highly similar RING‐CH domains shared by MARCH1 and MARCH8 E3 Ub ligases. Analysis to date, however, shows that *Ubl3^−/−^* mTEC or cTEC do not display significantly altered MHC II surface expression, and the conventional CD4^+^ T cell repertoire in naïve *Ubl3^−/−^* mice is unaltered [6]. To undertake a more detailed investigation of a role for UBL3 in thymic CD4^+^ T cell selection, here we have specifically addressed whether UBL3 functions with CD83. We show that UBL3 plays a role in CD83‐mediated selection of CD4^+^ T cells and identify specific stages of CD4^+^ T cell selection where CD83 and UBL3 are participating in this response.

## 2 Results

### 2.1 UBL3 Participates in CD83‐Mediated Regulation of Surface MHC II Expression in cTEC and mTEC

**FIGURE 1:**
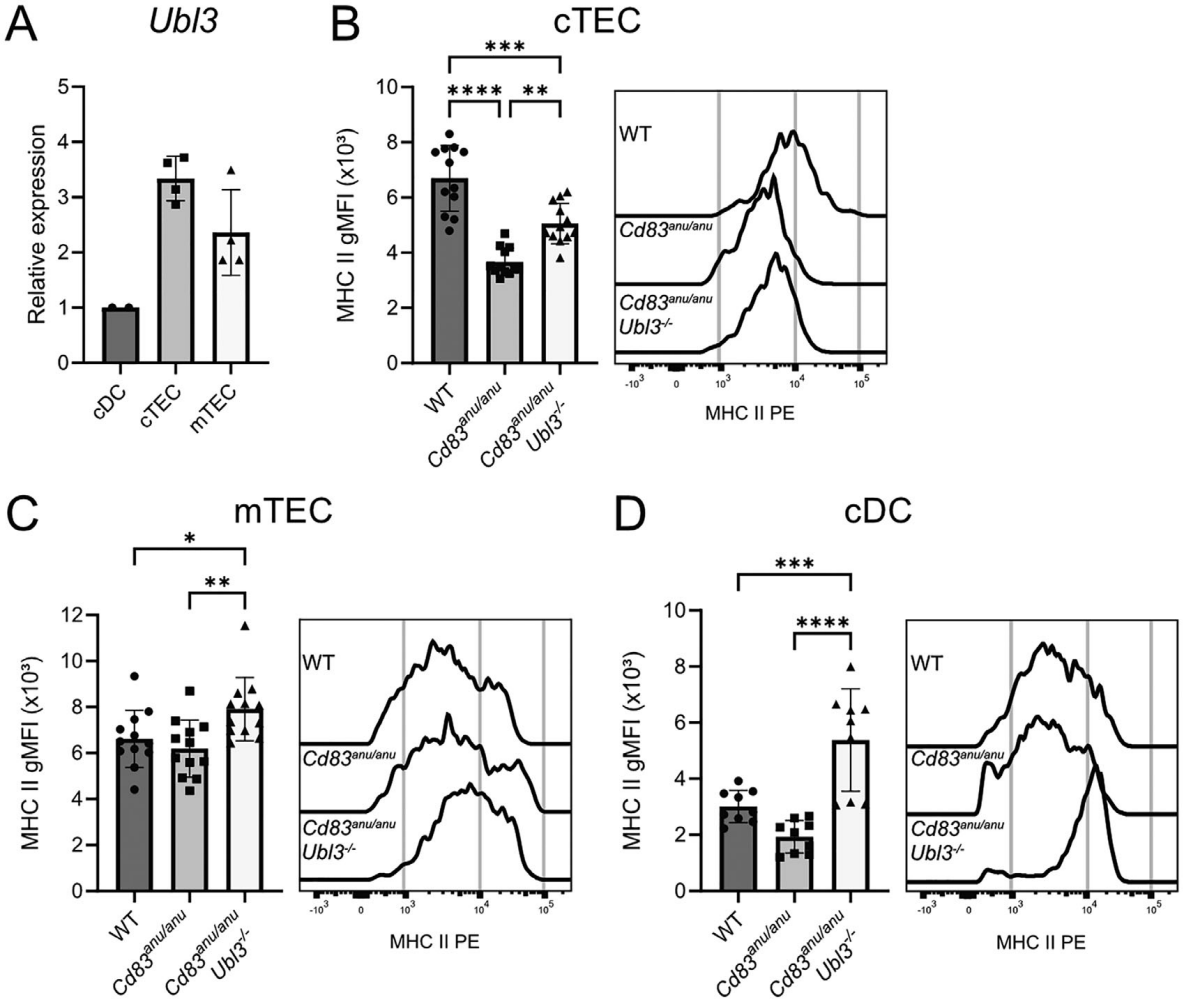
UBL3 participates in CD83‐mediated regulation of MHC II on the surface expression of thymic epithelial cells. (A) Relative expression of *Ubl3* compared with *Hprt* in WT splenic cDCs, cTECs, and mTECs. Data from two independent experiments. Each symbol represents one spleen or six pooled thymi. MHC II expression on the surface of enriched **(B)** cTECs and **(C)** mTECs from WT, *Cd83^anu/anu^*, and *Cd83^anu/anu^Ubl3* ^−^ *^/^* ^−^ mice was assessed by flow cytometry. Histograms are representative of four independent experiments. **(D)** MHC II expression on the surface of enriched thymic cDCs from WT, *Cd83^anu/anu^*, and *Cd83^anu/anu^Ubl3* ^−^ *^/^* ^−^ mice was assessed by flow cytometry. Histograms are representative of three independent experiments. **(B–D)** Each symbol represents one mouse, with data pooled from four independent experiments. Bars indicate mean ± SD. *p < 0.05, **p < 0.01, ***p < 0.001, ****p < 0.0001; one‐way ANOVA followed by Tukey's multiple comparison test.

To investigate a potential role for UBL3 in thymic selection, its expression pattern in the major thymic antigen presenting cell subsets was examined by quantitative real‐time PCR. Populations were purified using flow cytometry from wild type (WT) thymi and defined as CD11c^+^ MHC II^+^ cDC, CD45^−^EpCAM^+^MHC II^+^Ly51^+^ cTEC, and CD45^−^EpCAM^+^ MHC II^+^ UEA‐1^+^ mTEC. *Ubl3* was expressed at high levels by cTEC and mTEC relative to thymic cDC (Figure 1A). Given that UBL3 was detected as expressed in TEC, *Ubl3^−/−^* and *Cd83^anu/anu^* mice were intercrossed to investigate a role for UBL3 in CD83‐dependent CD4^+^ T cell selection. Thymic cellularity, mTEC, cTEC, and cDC proportions and numbers were unaltered in *Cd83^anu/anu^* or *Cd83^anu/anu^Ubl3^−/−^* relative to WT mice (Figure S1). To assess the contribution of UBL3 to MHC II plasma membrane turnover, MHC II surface expression was examined by flow cytometry. In *Cd83^anu/anu^* cTEC (Figure 1B), but not *Cd83^anu/anu^* mTEC (Figure 1C), levels of MHC II at the cell surface were significantly reduced compared with WT. This likely reflects CD83 expression in cTEC but not mTEC [5, 7]. Moreover, we and others have shown that decreased MHC II in the absence of CD83 likely stems from excessive MHC II ubiquitination by MARCH8 under conditions where CD83 is no longer able to impede MARCH8 activity [4, 5]. Deletion of *Ubl3* in *Cd83^anu/anu^* cells elicited a small, but significant rescue of MHC II surface expression by *Cd83^anu/anu^Ubl3^−/−^* cTEC relative to *Cd83^anu/anu^* cells (Figure 1B). In mTEC, a small increase in MHC II surface expression in *Cd83^anu/anu^Ubl3^−/−^* mTEC relative to *CD83^anu/anu^* and WT cells was also detected (Figure 1C). As we have previously reported [6], UBL3 was active in thymic cDC as evidenced by significant increases in MHC II levels in *Cd83^anu^* ^/a^ *^nu^Ubl3^−/−^* cDC relative to *Cd83^anu/anu^* and WT thymic cDC (Figure 1D). These data suggest that UBL3 is active in TEC, where it has a limited role in regulating MHC II surface expression under conditions where CD83 is impaired.

### 2.2 UBL3 Plays a Minor Role in the Development of the CD4^+^ Conventional T Cell and Regulatory T (Treg) Repertoires

**FIGURE 2:**
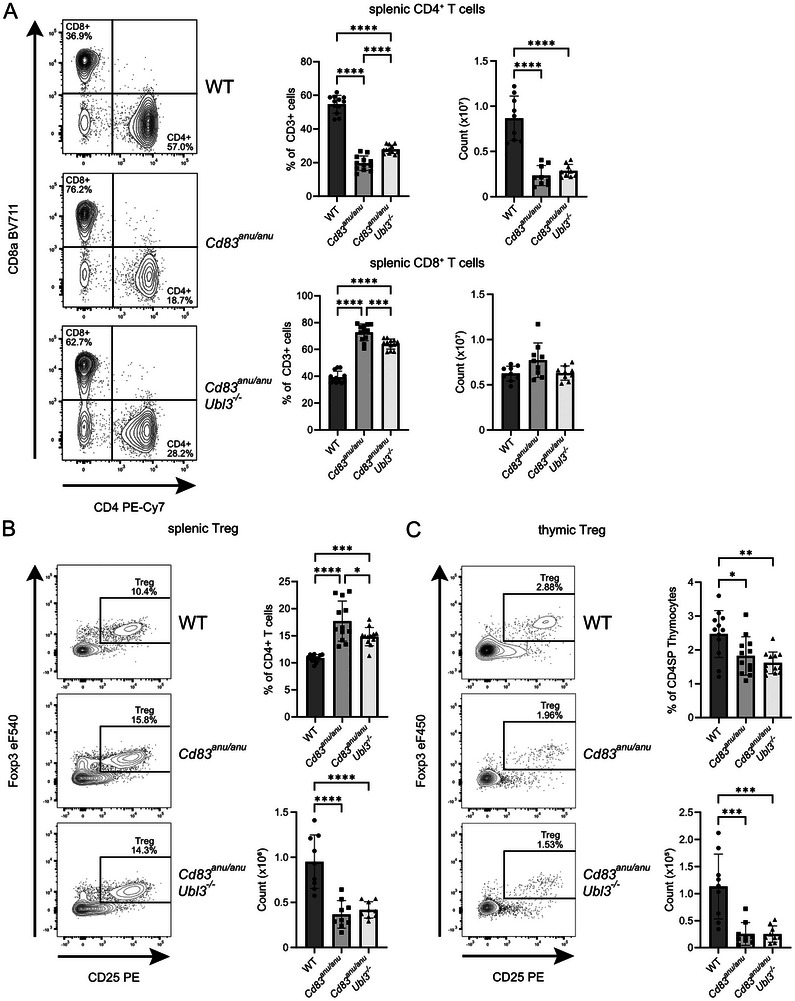
UBL3 has a minor role in the development of peripheral CD4^+^ T Cell and Treg populations. (A) Representative flow cytometry contour plots showing splenocytes isolated from WT, *Cd83^anu/anu^*, and *Cd83^anu/anu^Ubl3* ^−^ *^/^* ^−^ mice stained with CD4 and CD8 antibodies. Graphs show the number and proportion of splenic CD4^+^ T cells and CD8^+^ T cells. **(B)** Representative flow cytometry contour plots showing splenocytes isolated from WT, *Cd83^anu/anu^*, and *Cd83^anu/anu^Ubl3* ^−^ *^/^* ^−^ mice gated on CD3^+^ CD4^+^ T cells and stained with CD25 and FoxP3 antibodies. Graphs show the number and proportion of CD25^+^ Foxp3^+^ CD4^+^ Treg cells. **(C)** Representative flow cytometry contour plots showing thymic cells isolated from WT, *Cd83^anu/anu^*, and *Cd83^anu/anu^Ubl3* ^−^ *^/^* ^−^ mice, gated on CD4^+^ CD8^−^, and stained with CD25 and FoxP3 antibodies. Graphs show the number and proportion of thymic CD25^+^ Foxp3^+^ CD4^+^ Treg cells. **(A–C)** Contour plots are representative of three to four independent experiments. Each symbol represents one mouse, with data pooled from three to four independent experiments. Bars indicate mean ± SD. *p < 0.05, **p < 0.01, ***p < 0.001, ****p < 0.0001; one‐way ANOVA followed by Tukey's multiple comparison test.

CD4^+^ T cell selection in *Cd83^anu/anu^* versus *Cd83^anu/anu^Ubl3^−/−^* mice was assessed by analyzing conventional T cells in the spleen by flow cytometry. As we and others have previously described, *Cd83^anu/anu^* mice possess very few splenic CD4^+^ T cells [5, 6, 8, 9, 10]. A small rescue in the proportion of CD4^+^ T cells in the spleen was detected when comparing *Cd83^anu/anu^Ubl3^−/−^* to *CD83* ^*anu/anu*^ mice. Conversely, CD8^+^ T cell proportions were increased in *Cd83^anu/anu^* mice with a small decrease in *Cd83^anu/anu^Ubl3^−/−^* mice. Numbers followed a similar trend for CD4^+^ T cells (Figure 2A). For Tregs, an increase in the proportion of splenic Tregs was observed in *Cd83^anu/anu^* mice, with this increase being partially lost by the additional deletion of UBL3 (Figure 2B). For thymic Tregs, their proportion was reduced in both *Cd83^anu/anu^* and *Cd83^anu/anu^Ubl3^−/−^* mice, with no major difference between the two cohorts (Figure 2C). Overall, UBL3 plays a minor role in CD4^+^ T cell selection in the absence of CD83. This warranted a further examination of its role, together with CD83, in thymocyte development.

### 2.3 CD83 and UBL3 Participate in CD4^+^ T Cell Thymocyte Development

**FIGURE 3:**
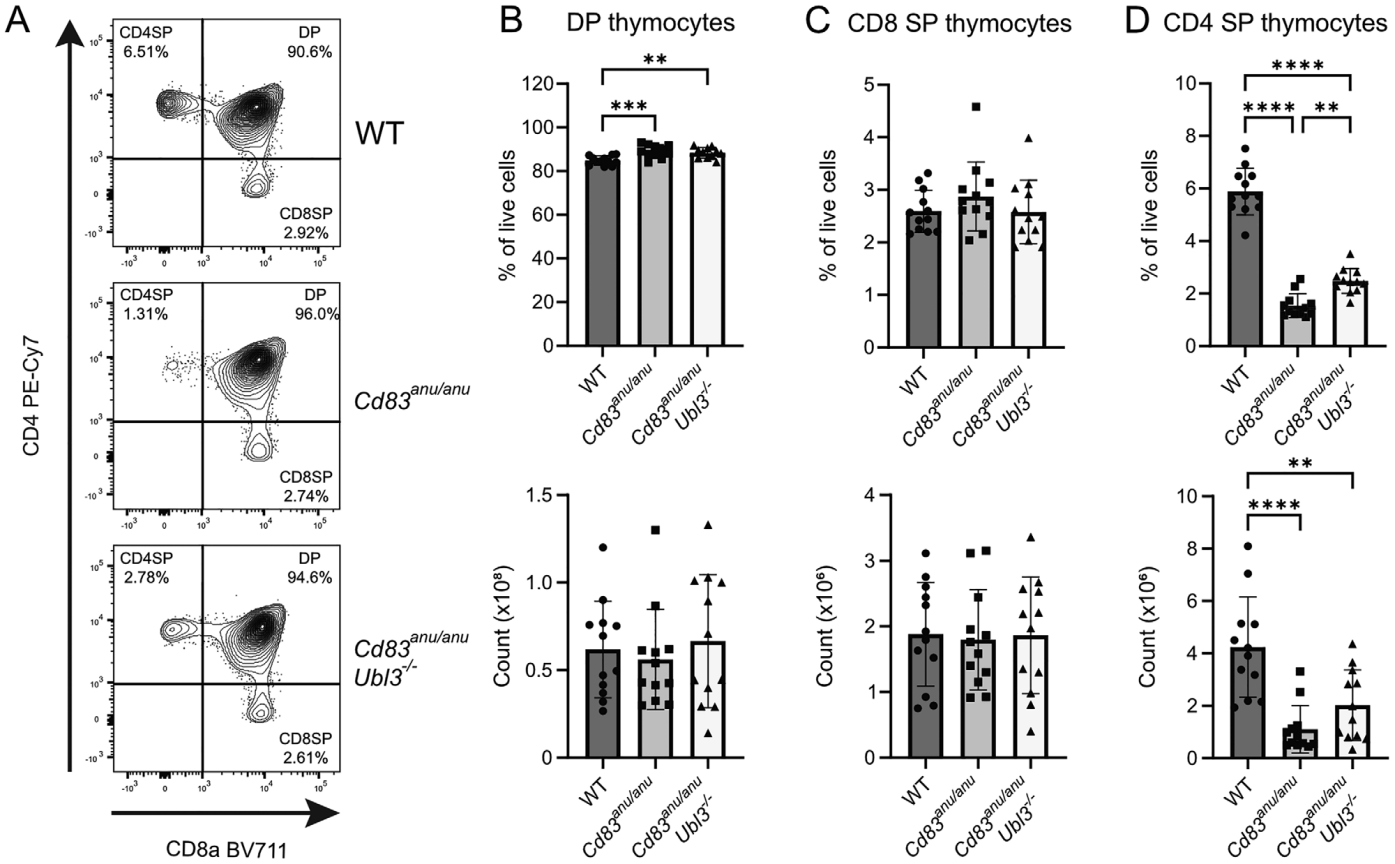
UBL3 has a minor role in the development of CD4 SP thymocytes. (A) Representative flow cytometry contour plots for thymocytes isolated from WT, *Cd83^anu/anu^*, and *Cd83^anu/anu^Ubl3* ^−^ *^/^* ^−^ mice stained with CD4 and CD8 antibodies. Number and proportion of **(B)** double positive (DP), **(C)** CD8 single positive (CD8 SP), and **(D)** CD4 single positive (CD4 SP) thymocytes from WT, *Cd83^anu/anu^*, and *Cd83^anu/anu^Ubl3* ^−^ *^/^* ^−^ mice. Each symbol represents one mouse, with data pooled from four independent experiments. Bars indicate mean ± SD. **p < 0.01, ***p < 0.001, ****p < 0.0001; one‐way ANOVA followed by Tukey's multiple comparison test. For **(B)**, one outlier was removed from each of the *Cd83^anu/anu^* and *Cd83^anu/anu^Ubl3* ^−^ *^/^* ^−^ groups.

To investigate CD83 and UBL3 in thymocyte development, specific thymocyte populations were examined by flow cytometry in the thymi of WT, *Cd83^anu/anu^*, and *Cd83^anu/anu^Ubl3^−/−^* mice. Analysis showed a small increase in the proportion (but not the number) of DP thymocytes (Figure 3A,B). CD8 SP thymocytes (Figure 3A,C) were unaltered in *Cd83^anu/anu^* and *Cd83^anu/anu^Ubl3^−/−^* mice compared with WT, while in contrast, CD4 SP thymocytes were reduced in *Cd83^anu/anu^* mice compared with WT, and the proportion and number were partially restored in *Cd83^anu/anu^Ubl3^−/−^* mice (Figure 3A,D).

**FIGURE 4:**
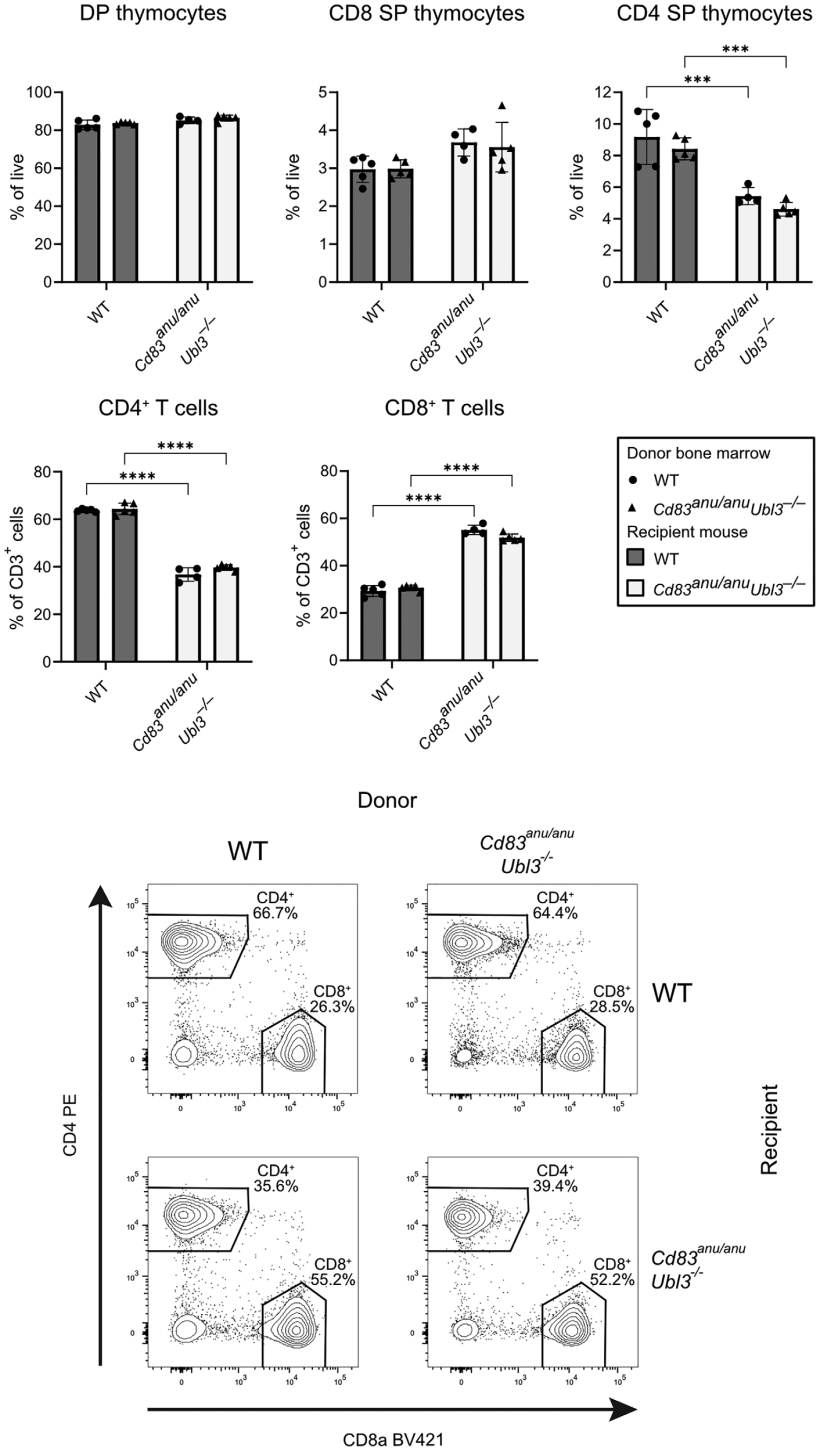
The non hematopoietic compartment is responsible for impaired CD4^+^ T cell development in *Cd83^anu/anu^ Ubl3* ^−^ *^/^* ^−^ mice. B6 WT (gray bars) or *Cd83^anu/anu^Ubl3* ^−^ *^/^* ^−^ (white bars) mice were lethally irradiated and reconstituted with WT Ly5.1 (circles) or *Cd83^anu/anu^Ubl3* ^−^ *^/^* ^−^ (triangles) bone marrow. Thymus and spleen were harvested at 6 weeks following reconstitution. The proportion of DP, CD4 SP, and CD8 SP thymocytes and CD4^+^ and CD8^+^ splenic T cells was determined by flow cytometry. Each symbol represents one mouse. Data from one experiment. Bars indicate mean ± SD. *p <0.05, **p <0.01, ***p <0.001, ****p <0.0001; two‐way ANOVA followed by Tukey's multiple comparison test.

Given that *Ubl3* deletion impacts MHC II expression in both thymic DCs and TECs, bone marrow chimeras were generated to delineate the respective contribution of these cell types to the observed CD4^+^ T cell selection defect. Bone marrow chimeras were generated using WT or *Cd83^anu/anu^Ubl3^−/−^* mice as hosts that were lethally irradiated and reconstituted with WT or *Cd83^anu/anu^Ubl3^−/−^* bone marrow. CD4^+^ T cell development was only reduced in *Cd83^anu/anu^Ubl3* ^−^ *^/^* ^−^ recipient mice and was unaltered when these mice expressed UBL3 and CD83 in their haemopoietic compartment (Figure 4). This suggests that UBL3 expressed in TEC, and not haemopoietic cells, is responsible for the observed CD4^+^ T cell phenotype.

**FIGURE 5:**
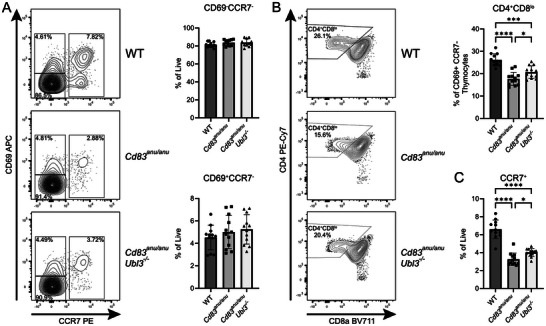
CD83 and UBL3 impact CD8 downregulation and CCR7 expression following TCR engagement by developing thymocytes. (A) Representative flow cytometry contour plots for thymocytes isolated from WT, *Cd83^anu/anu^*, and *Cd83^anu/anu^Ubl3* ^−^ *^/^* ^−^ mice, gated on CD4 SP, CD8 SP, and CD4^+^ CD8^+^ DP and stained with CD69 and CCR7 antibodies. Graphs show the proportion of CD69^−^CCR7^−^ and CD69^+^ CCR7^−^ thymocytes. **(B)** Representative flow cytometry contour plots for thymocytes isolated from WT, *Cd83^anu/anu^* and *Cd83^anu/anu^Ubl3* ^−^ *^/^* ^−^ mice gated on CD4 SP, CD8 SP, and CD4^+^ CD8^+^ DP CD69^+^ CCR7^−^ and stained with CD4 and CD8 antibodies. The graph shows the proportion of CD4^+^ CD8^low^ thymocytes of CD69^+^ CCR7^−^ thymocytes. **(C)** Medullary CCR7^+^ thymocytes of total live cells from WT, *Cd83^anu/anu^*, and *Cd83^anu/anu^ Ubl3* ^−^ *^/^* ^−^ mice. **(A–C)** Contour plots are representative of four independent experiments. Each symbol represents one mouse, with data pooled from four independent experiments. Bars indicate mean ± SD. *p < 0.05, **p < 0.01, ***p < 0.001, ****p < 0.0001; one‐way ANOVA followed by Tukey's multiple comparison test. For **(A)**, CD69^−^CCR7^−^ one outlier was removed from each of the *Cd83^anu/anu^* and *Cd83^anu/anu^Ubl3* ^−^ *^/^* ^−^ groups.

Further interrogation to determine the stages of thymocyte development where CD83 and/or UBL3 were playing a role in CD4^+^ T cell selection was undertaken. CCR7 and CD69 were used to define stages of thymocyte positive selection. CD69^−^ CCR7^−^ thymocytes are considered to be pre‐TCR engagement, while CD69^+^ CCR7^−^ and CCR7^+^ are considered to be thymocytes that have interacted with MHC molecules via their TCR [11, 12]. There was a trend for CD69^−^ CCR7^−^ thymocytes to be slightly elevated in the absence of CD83, and this increase was not altered by the loss of UBL3. CD69^+^ CCR7^−^ thymocytes were unaltered in either scenario (Figure 5A). Upon further examination, however, we observed CD8 downregulation following TCR engagement by CD69^+^ CCR7^−^ thymocytes, a key step in thymocyte lineage commitment following TCR‐MHC engagement [12, 13, 14], to be altered. A reduced proportion of CD69^+^ CCR7^−^ thymocytes had undergone CD8 downregulation in *C* *D* *83* *^anu/anu^* mice compared with WT, while in *Cd83^anu/anu^Ubl3^−/−^*, a small increase in CD8^low^ cells could be detected compared with *Cd83^anu/anu^* deletion alone (Figure 5B). CCR7^+^ medullary thymocytes, that have undergone positive selection, were significantly reduced in *Cd83^anu/anu^* thymi with a small, but significant, increase in *Cd83^anu/anu^Ubl3^−/−^* thymi (Figure 5C). Therefore, evidence of a role for UBL3 in CD4^+^ thymic selection could be detected in the thymocyte populations immediately downstream of TCR‐MHC II engagement.

**FIGURE 6:**
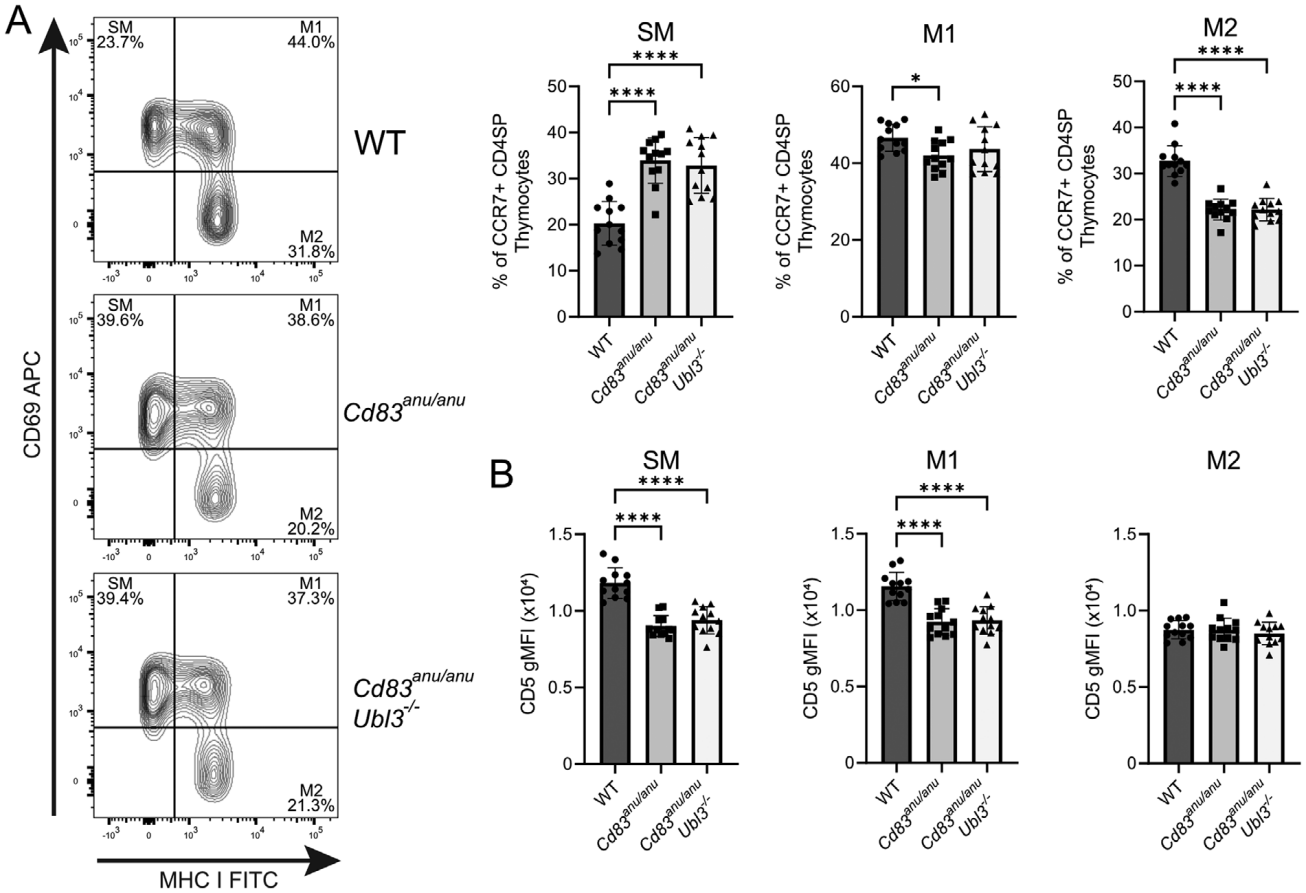
CD83, but not UBL3, impacts the development of positively selected CCR7^+^ CD4 SP thymocytes. (A) Representative flow cytometry contour plots for thymocytes isolated from WT, *Cd83^anu/anu^* and *Cd83^anu/anu^Ubl3* ^−^ *^/^* ^−^ mice, gated on CD4^+^ CCR7^+^ and stained with CD69 and MHC I antibodies. Graphs show proportion of CD69^+^ MHC I^low^ SM, CD69^+^ MHC I^high^ M1 and CD69^−^MHC I^high^ M2 medullary CD4 SP thymocytes from WT, *Cd83^anu/anu^* and *Cd83^anu/anu^Ubl3* ^−^ *^/^* ^−^ mice. **(B)** CD5 surface expression of SM, M1 and M2 medullary CD4 SP thymocytes from WT, *Cd83^anu/anu^*, and *Cd83^anu/anu^Ubl3* ^−^ *^/^* ^−^ mice. **(A, B)** Contour plots are representative of four independent experiments. Each symbol represents one mouse, with data pooled from four independent experiments. Bars indicate mean ± SD. *p < 0.05, **p < 0.01, ***p < 0.001, ****p < 0.0001; one‐way ANOVA followed by Tukey's multiple comparison test.

MHC I and CD69 expression were used to identify functionally distinct populations of semi mature (SM), mature 1 (M1), and mature 2 (M2) [15, 16] populations of CCR7^+^ CD4 SP thymocytes in WT, *Cd83^anu/anu^*, and *Cd83^anu/anu^Ubl3^−/−^* mice. SM, which are yet to develop T cell function, were elevated in *Cd83^anu/anu^* mice. In contrast, M2, CCR7^+^ CD4 SP thymocytes, which have gained some T cell activity, were reduced in *Cd83^anu/anu^* mice. M1, an intermediate between these two, was largely unaltered. For all populations, UBL3 deletion did not ameliorate the changes elicited by a lack of CD83 (Figure 6A). CD5 expression on each of these thymocyte subsets was also measured, given that its level is indicative of the strength of TCR‐MHC interaction [17, 18]. CD5 was reduced for *Cd83^anu/anu^* SM and M1 CCR7^+^ CD4 SP subsets relative to WT. Again, this was unaltered by UBL3 (Figure 6B). Together, this analysis suggested that while CD83 impacted the development of specific CCR7^+^ CD4^+^ thymocyte populations, UBL3 activity was not a major determinant at this later stage of CD4^+^ T cell selection.

## 3 Discussion

Here, we have identified that UBL3 participates in CD4^+^ T cell thymic selection, albeit in a minor role. Overall, our data suggest that UBL3 is active in TEC and participates in the early stages of CD83‐dependent positive selection of CD4^+^ T cells. This is demonstrated by the genetic deletion of UBL3 alleviating, but does not fully rescue, the impairment in CD4^+^ SP thymocyte and T cell development observed in the absence of functional CD83.

Extrapolation of our data that UBL3 can cooperate with CD83 in regulating TEC MHC II implies that UBL3 acts as an adaptor for MARCH8 E3 Ub ligase. The current model of UBL3 activity is that it promotes complexes containing MARCH1, E2 Ub ligase(s), and possibly other co‐factors and in doing so facilitates ubiquitination of specific substrates. Given that we have not directly examined TEC MHC II ubiquitination in the presence or absence of UBL3, and/or have not investigated a role for MARCH8 in the outcomes observed, this hypothesis remains speculative. While UBL3 regulates CD86 in haemopoietic cells, the lack of haemopoietic cell contribution to this response and the fact that we do not detect expression of CD86 by TEC [6, 19] suggests it is unlikely CD86 is involved. Another possibility is that the phenotypes observed are due to UBL3 exerting other functions in the (CD83‐deficient) thymus. UBL3 is a protein modifier [20] and is overexpressed in specific cancers [21, 22] and therefore, in addition to playing a role in CD83‐mediated regulation of MHC II TEC levels, it may be that there is an yet undescribed role for UBL3 in TEC or in the thymus.

It is well established that CD83 is required for CD4^+^ T cell development, a role attributed to its regulation of MHC II ubiquitination and turnover. Analysis of the defects in thymocyte and T cell development to date suggests CD83 is required for positive, but not negative, T cell selection [5]. In the absence of functional CD83, CD4^+^ T cell numbers in lymphoid organs are reduced, and those that develop exhibit defects in primary antibody responses, aberrant proliferation, and altered cytokine production as a result of reduced TCR, CD3, and CD5 [8, 9]. Here, we have more extensively characterized the specific defect in thymocyte development that occurs in the absence of CD83 and determined whether an impact on UBL3 can also be detected. Our analysis shows CD83, likely due to its perturbation of MHC II expression by cTECs, impacts specific events in the positive selection of CD4 SP thymocytes. Loss of CD83 function leads to a specific reduction in downregulation of CD8 by CD4^+^ CCR7^−^ thymocytes, a key step in CD4^+^ T cell lineage commitment. This step of thymic selection is also impacted by UBL3. Reduced SM and M2 CCR7^+^ CD4 SP thymocytes are also detected in the absence of functional CD83, despite these later stages of thymocyte development being independent of TCR‐MHC II engagement [16]. Therefore, these later defects are likely attributed to a reduction in thymocyte fitness that occurs in the populations immediately post‐TCR engagement. At these later stages, the impact of UBL3 is not detected.

In summary, here we report an in‐depth analysis of UBL3 and CD83 and their respective contributions to different stages of CD4^+^ T cell selection. A role for UBL3 was identified in the early stages of this response, highlighting that UBL3 is active in the thymus and contributes to the selection of CD4^+^ T cells.

## 4 Materials and Methods

### 4.1 Animals

C57BL/6J, *Cd83^anu/anu^* [10] and *Cd83^anu/anu^Ubl3^−/−^* mice were bred and maintained in specific pathogen‐free conditions at the Bio21 Molecular Science and Biotechnology Institute. *Cd83^anu/anu^Ubl3^−/−^* mice were generated by crossing *Cd83^anu/anu^* and *Ubl3^−/−^* [6]. The mice analyzed were 8–12 weeks old. Experimental procedures were approved by the Animal Ethics Committee of the University of Melbourne (21410).

### 4.2 DC Isolation

Primary splenic and thymic DCs were isolated as previously described [23]. Organs were finely chopped and digested with DNase I (Roche) and collagenase type III (Worthington). Cell clusters were disrupted by the addition of 10 mM EDTA. Light‐density cells were isolated by density‐gradient separation in 1.077 g/cm^3^ Nycodenz. Upper fractions were harvested and washed for analysis by flow cytometry or sorting. Splenic cDC preparations were stained with CD11c (N418, Biolegend, BV711) and MHC II (M5/114.15.2, WEHI Antibody Facility, PE) and sorted as CD11c^+^ MHC II^+^ using a BD Influx cell sorter for use in qPCR experiments. Thymic cDCs were stained with MHC II (M5/114.15.2, WEHI Antibody Facility, AF700), NK1.1 (PK136, BD Biosciences, PE), CD11c (N418, BV510), CD11b (M1/70, PE‐Cy7), and B220 (RA3‐6B2, PE) (all Biolegend) before acquisition with a BD LSR Fortessa flow cytometer. Data were analyzed using FlowJo software.

### 4.3 Thymic Epithelial Cell Isolation

TECs were isolated as previously described [24]. For flow cytometry analysis, single thymi were finely chopped and gently aspirated to deplete thymocytes. The thymocyte fraction was retained for cell counts. Remaining tissue fragments were digested with 0.1% DNase I (Roche) and 0.5 U/mL Liberase TM (Roche). Absolute cell numbers were obtained using an internal microsphere counting standard (BD Biosciences). Cell suspensions were stained with antibodies specific for MHC II (M5/114.15.2, WEHI Antibody Facility, PE), CD45 (30‐F11, PerCP), CD326 (G8.8, APC‐Cy7), Ly51 (6C3, FITC) (all Biolegend), and UEA‐1 (Vector Laboratories, biotinylated, streptavidin PE‐Cy7) before acquisition with a BD LSR Fortessa flow cytometer. Data were analyzed using FlowJo software.

For cell sorting, six pooled thymi were finely chopped and gently aspirated to deplete thymocytes. Remaining tissue fragments were digested with 0.1% DNase I (Roche) and 0.5 U/mL Liberase TM (Roche). TECs were enriched by negative selection. Cell suspensions were stained with anti‐CD45‐PE antibody before incubation with anti‐PE magnetic microbeads (Miltenyi Biotec). Cells were run through magnetic LS columns to remove CD45^+^ cells (Miltenyi Biotec). Flowthrough was collected and stained with antibodies specific for CD45 (30‐F11, PE), CD326 (G8.8, APC‐Cy7), Ly51 (6C3, FITC) (all Biolegend) and UEA‐1 (Vector Laboratories, biotinylated, streptavidin PE‐Cy7) before sorting with a BD Influx cell sorter.

### 4.4 T Cell and Thymocyte Analysis

Single spleens or thymi were processed into a single cell suspension and depleted of red blood cells using red cell lysis buffer. Thymic cell suspensions were stained with antibodies specific for CD4 (RM4‐5, PE‐Cy7 or GK1.5, PE), CD8 (53‐6.7, BV711 or BV421), CCR7 (4B12, PE), CD5 (53‐7.3, BV421), CD69 (H1‐2F3, APC) (all Biolegend) and MHC I (Y‐3, WEHI Antibody Facility, FITC). For splenic and thymic Treg analysis, single cell suspensions were stained with antibodies specific for CD3 (KT3‐1.1, spleen only, WEHI Antibody Facility, FITC), CD4 (RM4‐5, PE‐Cy7 or GK1.5 PE), CD8 (53‐6.7, BV711 or BV421), and CD25 (PC61, PE) (all Biolegend) before fixation and permeabilization with FoxP3 staining kit (eBioscience) and staining with anti‐FoxP3 antibody (FJK‐165, eBioscience, eFluor 450). CD45.1 (A20.1, PE‐Cy7, Biolegend) and CD45.2 (104, APC, Biolegend) were included for bone marrow chimera experiments. Absolute cell numbers were obtained using an internal microsphere counting standard (BD Biosciences). Data were acquired with a BD LSR Fortessa flow cytometer and analyzed using FlowJo software.

### 4.5 qPCR

RNA was isolated from sorted splenic cDCs, cTECs, and mTECs using the RNeasy Plus Micro kit (Qiagen). RNA was quantified using UV spectrophotometry (Eppendorf), and reverse transcription of RNA was undertaken using the sensiFAST cDNA synthesis kit (Bioline). cDNA samples were used as templates for the qPCR assay, using TaqMan Fast Advanced Master Mix (Life Technologies). Each sample was analyzed in triplicate. *Hprt* was used as an internal control. Relative gene expression was determined by Δ ΔCq = [Cq (sample) – Cq (HPRT)] – [Cq (reference)‐Cq (HPRT)] and fold change = 2^−ΔΔCq^.

### 4.6 Flow Cytometry

All antibody staining was undertaken in EDTA‐BSS + 2% FBS. CCR7 was stained at 37^0^C. Fixable Viability Dye eFluor 780 (eBioscience) staining was performed in PBS before antibody staining. DAPI and PI viability dyes were added to cell suspensions before acquisition. Gating strategies are presented in supplementary data (Figure S2).

### 4.7 Bone Marrow Chimera

C57Bl/6 or *Cd83^anu/anu^Ubl3* ^−^ *^/^* ^−^ were irradiated twice at 550 cGy (rad) three hours apart before being injected with 5 × 10^6^ bone marrow cells isolated from the tibia and femurs of donor B6.SJL‐Ptprca Pepcb/BoyJ (Ly5.1) or C57Bl/6 or *Cd83^anu/anu^Ubl3* ^−^ *^/^* ^−^ mice. Recipient mice were injected intraperitoneally with anti‐Thy1 mAb (T24, WEHI Antibody Facility) 18–24 h post irradiation to eliminate radio‐resistant host T cells. Drinking water was supplemented with neomycin (50 mg/mL, Enzo). Mice were reconstituted for 6 weeks before analysis.

### 4.8 Statistics

Statistical analyses were performed using GraphPad Prism software. Outliers that were more than 2.5 SD from the mean were removed from the analysis. Where outliers were removed, the details are indicated in the figure legend.

## Conflicts of Interest

The authors declare no conflicts of interest.

## Supporting information




**Supporting File**: eji70143‐sup‐0001‐SupMat.pdf.

## Data Availability

Data are available from the corresponding author upon reasonable request.
