# Supplementary material for: UBL3 Participates in the Early Stages of CD83‐Dependent CD4+ T Cell Selection
Source: Eur J Immunol. 2026 Feb 4;56(2):e70143. doi: 10.1002/eji.70143 (PMC12869844; doi:10.1002/eji.70143)
Supplement: Supplementary file 1 — Supporting File: eji70143‐sup‐0001‐SupMat.pdf. [file EJI-56-e70143-s001.pdf]

Supplementary Figure 1

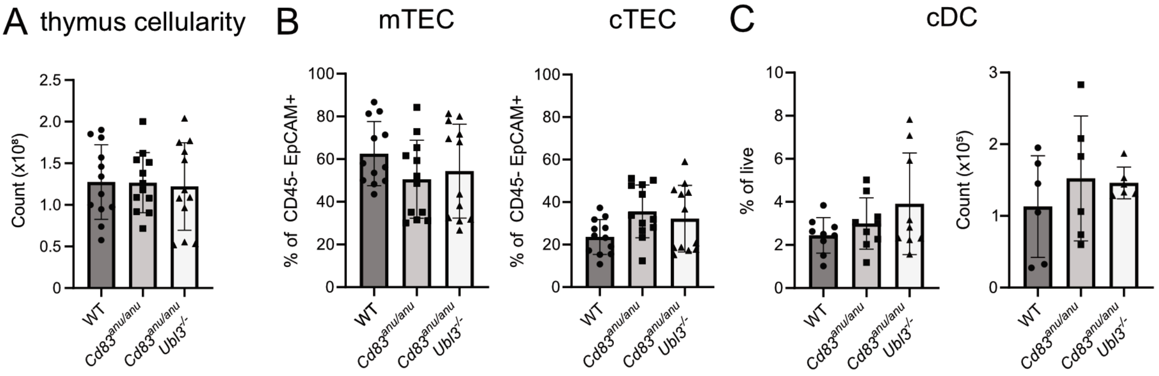

Supplementary Figure 2

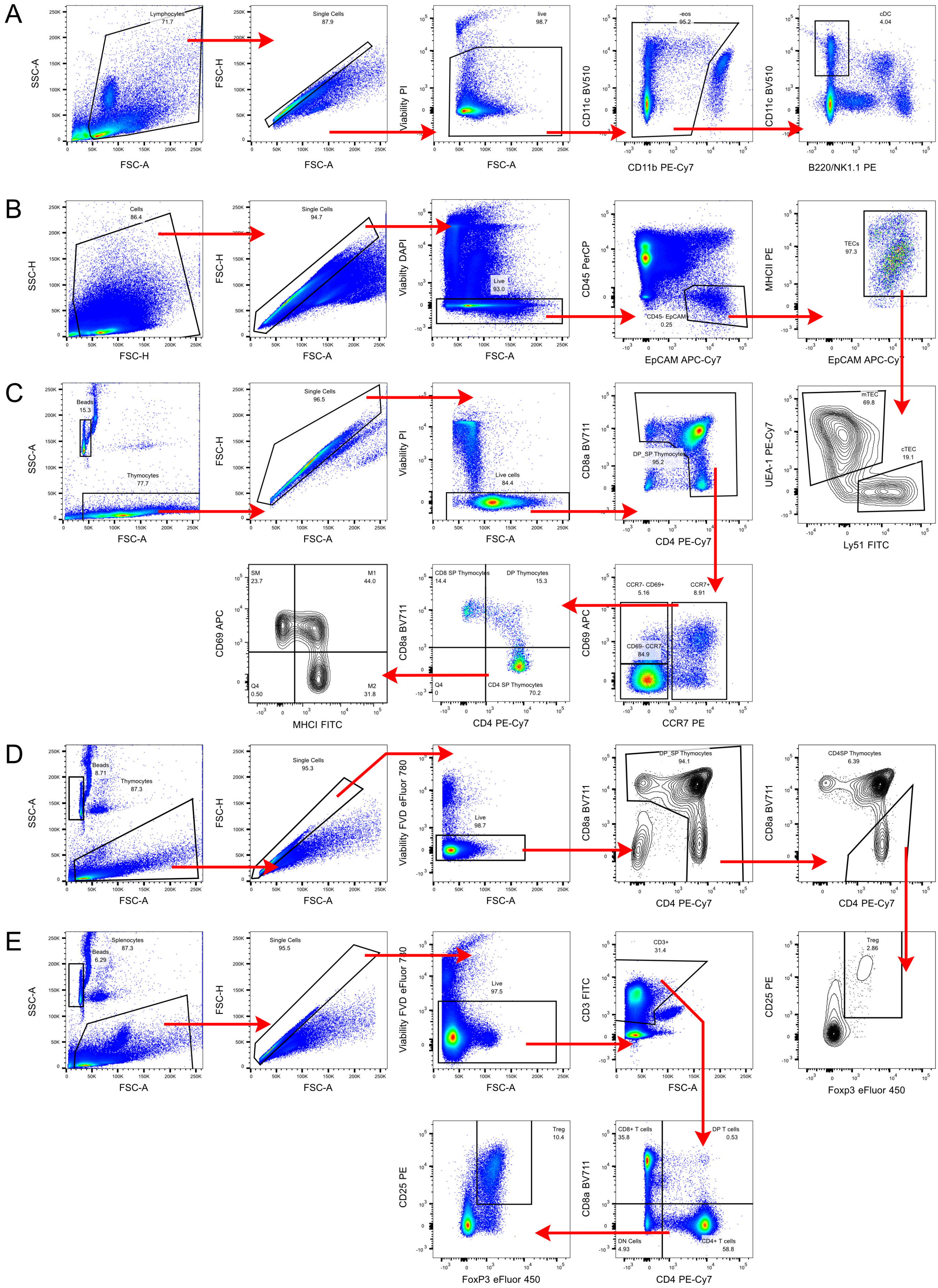

Supplementary Figure Legends

Supplementary Figure 1: CD83 and Ubl3 loss of function do not affect thymic epithelial cell or thymic cDC proportions. (**a**) Thymus cellularity of WT, $Cd83^{anu/anu}$ and $Cd83^{anu/anu}Ubl3^{-/-}$ mice. **(b)** Proportion of mTEC and cTECs in WT, $Cd83^{anu/anu}$ and $Cd83^{anu/anu}Ubl3^{-/-}$ mice. **(c)** Proportion and number of thymic cDCs in WT, $Cd83^{anu/anu}$ and $Cd83^{anu/anu}Ubl3^{-/-}$. Each symbol represents one mouse, with data pooled from **(c)** three or **(a, b)** four independent experiments. Bars indicate mean +/- SD.

Supplementary Figure 2. Gating strategy for flow cytometry analysis of cell populations analysed. Gating strategies for **(a)** thymic DCs, **(b)** TEC, **(c)** thymocyte development stages, **(d)** thymic and **(e)** splenic Tregs.
